# Evaluation of Pathogen Removal in a Solar Sludge Drying Facility Using Microbial Indicators

**DOI:** 10.3390/ijerph7020565

**Published:** 2010-02-12

**Authors:** Emily F. Shanahan, Anne Roiko, Neil W. Tindale, Michael P. Thomas, Ronald Walpole, D. İpek Kurtböke

**Affiliations:** 1 Faculty of Science, Health and Education, University of the Sunshine Coast, Maroochydore DC, 4558, Queensland, Australia; E-Mails: efs001@student.usc.edu.au (E.F.S.); aroiko@usc.edu.au (A.R.); ntindale@usc.edu.au (N.W.T.); 2 Sunshine Coast Water, 38 Commercial Road, Maroochydore, 4558, Queensland, Australia; E-Mails: Mike.Thomas@sunshinecoast.qld.gov.au (M.P.T.); Ron.Walpole@sunshinecoast.qld.gov.au (R.W.)

**Keywords:** biosolids, sewage sludge, microbial indicators, human pathogenic bacteria, solar dryer

## Abstract

South East Queensland is one of the fastest growing regions in Australia with a correspondingly rapid increase in sewage production. In response, local councils are investing in more effective and sustainable options for the treatment and reuse of domestic and industrial effluents. A novel, evaporative solar dryer system has been installed on the Sunshine Coast to convert sewage sludge into a drier, usable form of biosolids through solar radiation exposure resulting in decreased moisture concentration and pathogen reduction. Solar-dried biosolids were analyzed for selected pathogenic microbial, metal and organic contaminants at the end of different drying cycles in a collaborative study conducted with the Regional Council. Although fecal coliforms were found to be present, enteroviruses, parasites, *E. coli*, and *Salmonella* sp. were not detected in the final product. However, elevated levels of zinc and copper were still present which restricted public use of the biosolids. Dilution of the dried biosolids with green waste as well as composting of the biosolids is likely to lead to the production of an environmentally safe, Class A end-product.

## 1. Introduction

Sewage sludge is an inevitable by-product of wastewater treatment and with the present rate of population increase, the volumes of sludge to be dealt with will continue to grow, constituting a problem for local authorities. The agenda for global sustainability provides a strong mandate for waste streams, such as sewage sludge, to be converted into biosolids of various grades that can be used to restore degraded lands by reintroducing nutrients and soil conditioning agents (Table 1). However, the “solid fraction”, or biosolids component contains microorganisms, including some that are potentially harmful, toxic metals, macro- and micronutrients. As a result, the reuse of inadequately treated biosolids might present a recognized public health risk [1–3].

Specific characteristics of the biosolids depend upon the quality of the sewage sludge and the type of treatment processes performed [4]. There are many techniques used for attempted stabilization and disinfection of sewage sludge. Different methods include biological (anaerobic digestion, mesophilic and thermophilic aerobic digestion) and non-biological techniques (lime stabilization, composting, advanced alkaline stabilization, heat-produced pasteurization, air and heat drying, and treatment through constructed wetlands [5–7]. Anaerobic digestion, aerobic digestion, and lime stabilization produce, on average, Class B biosolids products [8] (Table 1). However, biosolids products applied to lawns and home gardens, either sold or given away in bags or other containers, must meet Class A requirements. As a result, alternative methods are now being implemented to disinfect and stabilize sewage sludge, such as solar drying [9].

By 2006, more than 70 solar drying installations were built in the European Union, the United States, and Australia [9]. Drying, in addition to anaerobic stabilization and mechanical dewatering, has been found to reduce the volume of remaining material. Also, as part of the drying process, most odor and pathogen problems are eliminated. The system appears to be superior to conventional heat drying processes, which are technically complex, require high investment and consume large quantities of both thermal and electrical energy [9].

In a collaborative study conducted by the University of the Sunshine Coast and Sunshine Coast Water, the efficiency of an evaporative solar dryer system was investigated under full-scale operating conditions. Microbial indicators were used to assess pathogen reduction in the final dried product. Heavy metal and moisture levels were also measured to ascertain whether the final product would meet the EPA (NSW) guidelines [8] for biosolids products for unrestricted distribution and land application.

## 2. Methods

### 2.1. Sampling Site: Evaporative Solar Dryer

**Figure 1.**
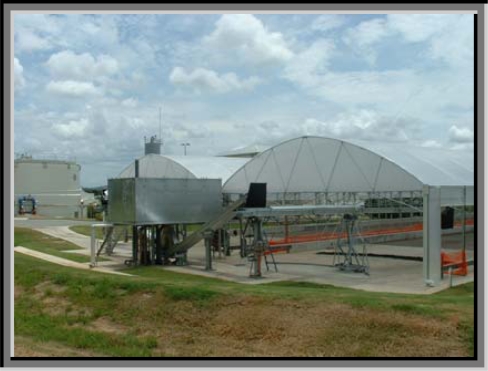
Maroochydore STP solar dryer.

The biosolids samples for analysis were obtained from a solar dryer located on the property of Maroochydore Sewage Treatment Plant (STP) about 100 km north of Brisbane, Queensland. Maroochydore STP treats an average flow of 24 ML/day. Currently the plant serves a population of approximately 95,000 people. The solar dryer consists of two sludge drying beds, which are positioned parallel to each other. Each of the sludge drying beds has an effective length of 106 m and a width of 13 m. The total drying bed surface area of the two drying beds is 2,756 m^2^. The side walls of the drying beds are 600 mm high; the typical depth of sludge within the drying beds is between 150 mm and 300 mm and the depth of the sludge at the inlet end of the drying bed is 250 mm. Roll-down clear plastic sheet walls are used to exclude rain without blocking solar radiation. The drying beds are operated in parallel as a continuous process (Figure 1).

The sewage sludge entering the solar dryer is of municipal origin deriving primarily from domestic sources, produced by households, mixed with sludge sourced from commercial and industrial works areas. The sludge entering the sludge drying beds consists of a mixture of fermented primary sludge, waste activated sludge, and alum sludge that has been anaerobically digested (Table 2).

**Figure 2.**
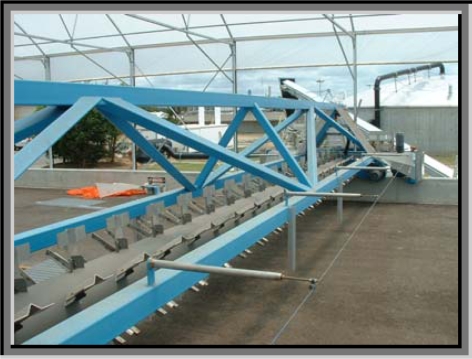
Mechanical sludge turner.

Both drying beds contain a sludge turning mechanism that travels backwards and forwards along the drying beds. The purpose of the sludge turning mechanism is three-fold: it turns over the sludge to enhance the rate of drying, it aerates the sludge, and it transports it along the drying beds from the inlet (wet) end to the outlet (drier) end. It takes about an hour for the sludge turner to complete one full cycle back and forth along each bed (Figure 2).

Sludge is, under current operating conditions, moved approximately 10 m along the drying beds each day; the average drying time is about 10 days. Once the biosolids material has reached the outlet end of the drying beds, it remains in a stockpile zone for an average of two days before being pushed by the turner mechanism onto a conveyor and loaded into trucks for transport from the site.

### 2.2. Sampling Strategy, Sample Collection and Analysis

The sampling was conducted three times in total during the study, for durations of several days each time, to reflect both ‘within-batch’ (spatial) and ‘between-batch’ (temporal) variation of sampling runs [10]. The first, preliminary sampling run was sampled over nine days as a trial of the experimental process (October 4^th^−12^th^, 2007). The subsequent two sampling runs had different durations: Sample Run I, lasted for 12 days (November 8^th^−19^th^, 2007) while Sample Run II, lasted for 18 days (January 18^th^−February 4^th^, 2008).

Samples were obtained from the drying beds every day during each sampling run. The locations from which the samples were collected were the wet end (inlet), 6 m, 26 m, 46 m, 66 m, 85 m, and the drier end (outlet)—105 m. Strips of rubber were added to the sewage sludge entering the drying beds as tracers, to indicate the relative position of the sludge to be sampled along the solar dryer. At each of the seven sampling sites down the length of the drying beds, two samples were obtained from a cross section of the bed to cater for small-scale variations. The biosolids holding areas at the outlet ends of the drying beds were sampled also. Since both beds were operating simultaneously, the samples from each drying bed were combined, forming one composite sample for each sampling distance. However, during Sample Run II, mechanical problems with the operation of Bed 1 resulted in the collection of samples from Bed 2 only until the end of the sampling period.

The samples were collected and stored in sterile screw-cap containers and were transported on ice, stored below 4 °C and processed within 24 hours [11]. Moisture content, pH, and temperature of the samples were also measured.

In order to determine the moisture content of the biosolids, approximately 10 g of each of the homogenized, composite samples were heated for 12 hours at 105 °C [12,13]. They were then placed in a Desiccator and cooled to room temperature before re-weighing. The biosolids were then placed back in the oven for one hour and re-weighed after cooling. If the post-drying weight was within 1% of the previous weight, the sample was considered to have reached a plateau of moisture concentration [13]. The pH of the samples was measured by a pH meter (Radiometer) by adding one gram of sample to 20 mL DI water; the mixture was then agitated using a flask shaker for 20 min at 500 osc/min [14]. Temperature profiles were determined on two different days for Bed 2 during Sample Run II.

Analyses were conducted in accordance with the procedures outlined in the EPA (NSW) Guidelines (1997) [8] and compared to the biological standards for initial process verification and stabilization for Grade A biosolids (Table 3).

Levels of selected metal and organic contaminants in the final product were also measured as per the EPA (NSW) Guidelines (1997) [8] and compared to those considered acceptable for each grade of biosolids, ranging from A to D (Table 4).

### 2.3. Environmental Variables

Several potentially confounding environmental variables were recorded during the sampling periods, and included atmospheric pressure, humidity, rainfall, solar exposure, and temperature. These data were provided by the Australian Bureau of Meteorology Station (040861) located at the Maroochydore Airport, about 5km from the STP. Local wind direction and speed were measured on-site at the Maroochydore STP.

### 2.4. Biological Analysis

#### 2.4.1. Bacteria

One gram of each biosolids sample was mixed with 99mL of sterile, deionized (DI) water in sterile 150 mL Schott bottles to make a 10^−2^ dilution (weight/volume). The sample was shaken using a mechanical shaker at 600 osc/min for 20 min and serial 10-fold dilutions were performed. Aliquots of 250 μl from selected dilutions were later transferred onto appropriate media to test for the presence of indicator microorganisms listed in Table 3. Fecal coliforms were analyzed using MacConkey Agar 3 (Oxoid Ltd.) [15] and *Salmonella* sp. and *Shigella* sp. were analyzed using *Salmonella/Shigella*® agar (Bio-Rad Laboratories Inc.) [16]. Highly selective medium UriSelect®4 was also used to detect the presence of *Candida albicans*, *Enterobacter cloacae*, *Enterococcus fecalis*, *Klebsiella pneumoniae*, *Proteus mirabilis*, *Pseudomonas aeruginosa*, *Shigella* sp., *Staphylococcus aureus*, *Staphylococcus saprophyticus*, and *Streptococcus agalactiae* [17].

All plates were incubated for 48 h ± 2 h at 37 °C. Following incubation, colony forming units (cfu) were counted and selected colonies were further subjected to confirmatory analyses using routine biochemical tests (citrate utilization, hydrogen sulphide, indole production, methyl red, motility, urease, and Voges-Proskauer) [18].

#### 2.4.2. Bacteriophages and Enteric viruses

Bacteriophages were targeted as indicators of viral survival throughout the drying process [1,24,26] One gram of each composite sludge sample was added to a 250 mL flask containing 20 mL of Tryptic soya broth (TSB) (Difco) seeded with a loopful of a laboratory strain of *E. coli* (JM109) as a baiting host for phages. The resulting suspensions were then incubated at 37 °C in a temperature-controlled shaker (Brunswick) for 12 hours at 600 rpm. The suspension was then centrifuged at 2,500 rpm for 20 min and the resulting supernatant was filtered through a 0.22 μm membrane filter using a suction filter unit (Stericup, Micropore Corporation, USA). Filtered samples were then assayed for the presence of bacteriophages using an *E. coli* (JM109) host strain inoculated onto peptone yeast extract calcium (PYCa) agar plates. The plates were incubated at 37 °C and monitored for plaque formation [19]. Subsamples were sent at a later date to an external accredited laboratory to confirm the presence of enteric viruses.

#### 2.4.3. Determination of Helminth ova: *Ascaris* sp. and *Taenia* sp

Twenty grams of the biosolids samples were dissolved in a buffer and a flotation method was used to recover helminth ova [1]. Identification was conducted at an external independent NATA accredited laboratory.

#### 2.4.4. Determination of metal/metalloid and organic contaminants

Composite samples of the final biosolids product for Sample Runs I and II were sent to an external NATA accredited laboratory for metal, metalloid and organic analysis.

## 3. Results

Bacteriophages were detected during the first several days of sampling but not thereafter (Table 5). The enteroviral analysis results were consistent with this trend in bacteriophages as the they were present at the start of the solar drying treatment and were not detectable in the final samples. In addition, no helminth ova were present in the final samples (Table 5).

Fecal coliforms (FCs) were present on the final day of both sampling runs; with higher numbers observed at the end of Sample Run II when compared to Sample Run I. *E. coli* and *Salmonella* sp. however, were not detected (Table 6).

The bacterial analysis revealed conflicting results. The chromogenic identification on the UriSelect®4 agar indicated the presence of *Candida albicans, Enterobacter cloacae, Enterococcus fecalis, Klebsiella pneumoniae, Proteus mirabilis, Staphylococcus aureus* and *Streptococcus agalactiae* (Table 7). However, the follow-up biochemical tests conducted on the isolates obtained from UriSelect®4 agar indicated the likelihood of different species altogether (Tables 8).

Selected biosolids-derived isolates were further tested against 19 different antibiotics and resistance was exhibited towards some of the antibiotics (Table 9).

A total of 22 organic contaminants and 9 metals/metalloids were analyzed from both sample runs (Table 10). Determination of whether the samples met the EPA (NSW) guidelines (1997) [8] generally depended on levels of metals. Metal concentrations were low enough to meet at least Grade C criteria in all samples. During runs, Grade A criteria were met for final concentrations of arsenic, cadmium, chromium, lead, nickel, and selenium. For the final mercury concentrations, the Grade A criterion was met for Sample Run I and just narrowly missed the Grade A threshold for Sample Run II. Final zinc concentrations were measured within Grade B standards for both Sample Runs I and II, whereas final copper concentrations only met the Grade C criterion.

The pH of each sample was not found to fluctuate significantly from around neutral pH. The pH ranged between 6.36 and 7.42 (average: 6.98) during Sample Run I and between 6.57 and 7.12 (average: 6.96) during Sample Run II.

The performance of the solar dryer was known to be affected by the weather conditions and a record of weather conditions was kept during the study. During the experiments, humidity ranged between 49% and 88%, with an average of around 65%−75%. Outside temperatures ranged between 13 °C and 29 °C, with an average during the day of 24 °C−26 °C. Wind direction was mainly from the south-east with periods blowing from the north-east over both sampling periods. The wind speed ranged between 13 km/h and 28 km/h (average: 21 km/h).

During Sample Run I, there were four days of rainfall (Days 3, 9, 10, and 11) over the course of the 12-day sampling period. The highest rainfall recorded during this period was 2.6 mm and the lowest was 0.4 mm. While the solar dryer was covered, periods of cloud cover, rain and cooler temperatures would have affected the performance of the drier. There were 10 days of rainfall during the 18 day Sample Run II. The highest rainfall recorded during this period was 26.0 mm on Day 18 and the lowest was 0.2 mm.

Samples of the biosolids entering the solar dryer at Maroochydore STP were found to have a moisture content of approximately 80% with an average temperature of around 35 °C−40 °C. The moisture content of the biosolids product dropped from 80% to 58% during the 12 day period of Sample Run I and the moisture content during the 18 day period of Sample Run II dropped from 81% to 30%. In the final sample from the 2^nd^ run, a temperature of 67.5 °C was recorded within the sludge. Such an elevated temperature indicated that composting had begun in the “dried” final biosolids product waiting to be removed from the solar dryer and only thermophilic and thermotolerant microorganisms would thrive. This was an unexpected result.

## 4. Discussion

The EPA (NSW) guidelines (1997) [8] set health-based criteria for specific contaminants in biosolids intended for reuse under the following categories: potentially pathogenic bacteria, enteric viruses, parasites, heavy metals and organics. Such criteria are required because the reuse of inadequately processed sludge may pose risks to public health. Nevertheless, the accurate assessment of treated biosolids against such guidelines has been recognized as problematic due to the complex nature of pathogen detection in environmental matrices such as biosolids [20]. In response, one objective of this study was to trial a rapid screening technique to select potentially pathogenic microbes. The concept of ‘screening’ was applied in the sense that the technique used was rapid and did not require extensive biochemical or molecular characterization.

UriSelect®4 is a selective agar for ten different species of potentially pathogenic microorganisms. UriSelect®4 has been validated previously for isolation and enumeration of these pathogens in clinical samples, but not in environmental samples at the time of this study [21]. During the course of sampling and analysis, spurious results were observed that placed the reliability of this method into question. For example, the selective media indicated apparently high concentrations of *Staphylococcus aureus*, a Gram-positive bacterium, in numerous samples of the treated biosolids, which was unexpected on the basis of previous studies. Rusin *et al*. [20] found that, while *S. aureus* could be detected in raw sewage sludge, it is very unlikely to be found in samples of biosolids or biosolids aerosols. During the present study, the colonies indicating the presence of *S. aureus* chromogenically were found to be Gram-negative bacteria when Gram-stained. This suggests that, chromogenic identification of isolates on UriSelect®4 led to misclassification of bacterial species from the biosolids samples. Similar results were obtained for yeast and other bacterial species. Consequently bacterial isolates from subsequent sampling runs were characterized further using biochemical tests alongside the UriSelect®4 analysis.

It is interesting to note that the UriSelect®4 media were not designed to promote the growth of microorganisms that were found, upon biochemical characterization, to belong to the genera *Morganella*, *Providencia*, *Rahnella*, and *Yersinia*. Despite this, the lack of precision by UriSelect®4 agar for chromogenic species determination in this environmental context seems to rule it out as an appropriate screening technique for the selectivity of microbial populations in sewage sludge and biosolids, particularly when samples need to be assessed against health-based guidelines.

Another key microbial indicator used to assess the microbiological quality of biosolids, *Salmonella* spp. were not detected in post-treatment samples when using both *Salmonella*/*Shigella*® agar and biochemical characterization. The lack of *Salmonella* spp. could have been due to competition between microflora in compost samples [21] or to desiccation, which would be expected to affect any species not forming endospores. Culture-independent techniques such as DNA probe test kits for salmonellae [22], may have indicated their presence, but would not have been able to distinguish between live, infectious pathogens, and dead, or non-infectious, pathogens [1].

The EPA (NSW) guidelines (1997) [8] for biosolids also stipulate acceptable levels of fecal coliforms (FCs). These bacteria were detected in the final biosolids products. However, there are well-known disadvantages of using fecal coliforms as indicator organisms in public health studies [23]. For example, upon exposure to disinfection processes, viruses and protozoan cysts have been found to be more capable of survival when compared to FCs [23].

Presence/absence detection of bacteriophages was used as a surrogate measure for the presence of enteric viruses using an *E. coli* JM109 strain as a baiting host. In this study, bacteriophages specific to this host were present initially but undetectable from approximately days 5 and 6 onwards. Enteroviral results obtained from an outside accredited laboratory showed that high levels were present prior to treatment in the solar dryer and were below EPA (NSW) Guidelines (1997) [8] in samples collected at the end of the drier. The consistent correlation between the actual data for enteric viral presence and presence of bacteriophages specific to the host strain of *E. coli* JM109 used in this study might suggest that (i) that the solar drying treatment was effective in removing enteric viruses and (ii) that the use of target specific bacteriophages, as a surrogate of enteric viruses was appropriate. Such findings also agree with other bacteriophage studies in sludge treatment systems [1,24,25]. However, as suggested by Lucena *et al*. [26] results obtained from one phage group should not be extrapolated to another.

Both copper and zinc were present in concentrations above the EPA (NSW) Guidelines (1997) [8] criteria for Class A requirements. Municipal and industrial sources of wastewater are combined at Maroochydore STP, but the likely sources of copper and zinc present in the wastewater are from corrosion of pipes and plumbing components and from run-off from building roofs (pers. com., Maroochydore STP staff). Low heavy metal content has been achieved in other regions: for example in Singapore, due to separation of municipal and industrial wastewaters [27]. The elevated concentrations of copper and zinc, and their sources, in the Maroochydore STP biosolids requires further investigation. Dilution with additional, low metal content, organic wastes could achieve acceptable heavy metal levels in the dried biosolids.

Solar radiation can impact on some microorganisms. However, the biosolids at Maroochydore STP are not directly exposed to solar radiation as the drier is covered by a plastic film roof that has walls that can be lowered in blustery, wet weather. Nearly all infrared, some visible and only the longer wavelength UV rays will pass through the plastic roof of the dryer. Previous studies have shown that it is the short wavelength UV light that is most effective for killing pathogenic microorganisms [28]. The short wavelength UV light is blocked by the roof of the solar dryer so the microbial die-off mechanisms are unlikely to be driven by exposure to UV energy. This is important to note as fecal coliforms have been found to be the most sensitive microorganisms to sunlight when compared with enterococci and phages [28].

Previous studies have found the following factors to be influential in the rate of water removal from sewage sludge: drying is a function of (1) the dewatering device or technology used, (2) the operational parameters of the device, (3) chemical additives used to condition the solids for more effective solid-liquid separation, (4) the properties of the solids entering the process, (5) physical pre-treatment prior to conditioning, and (6) maintaining certain characteristics of the solids prior to dewatering. Currently, no chemical additives are added to the sludge to enhance drying at Maroochydore STP, although polyelectrolyte is dosed prior to the dewatering centrifuge, and consequently the drying process is physical.

The moisture content of sewage sludge-derived biosolids is a major factor in biosolids-related expenditure: the heavier the sludge, the more it costs to transport. One of the purposes of the solar dryer is to reduce moisture content in the sludge and this was the case at the Maroochydore STP solar dryer. Based on previous studies undertaken on similar models of solar dryers, the prime predictors of evaporation consisted of (1) outdoor solar radiation, (2) outdoor air temperature, and (3) ventilation flux, if applicable [9].

From the profile of temperatures measured within the sludge, at varying locations on the solar dryer, it appeared that temperatures were high enough to promote evaporation of water and subsequent drying but not high enough to directly impact on pathogenic organisms. However, the temperature readings from the biosolids that were stockpiled at the end of the drying beds were elevated, at approximately 70 °C; high enough to indicate the likelihood that composting was occurring and for bacterial levels to be reduced. This was an unexpected result and would depend, presumably, on how frequently the dried sludge was removed from the end of the dryer. To improve the removal of pathogenic organisms, composting could be included as part of the overall process. This might be important in reducing the numbers of antibiotic resistant bacteria which were found to survive at the end of the drying process. In addition, mixing with other organic wastes could be used to enhance composting, and additionally, to dilute and lower heavy metal concentrations at the same time, improving the overall suitability of the dried and composted biosolids for use as a soil conditioner.

## 5. Conclusions

The results of this study demonstrate that the dried biosolids product from the Maroochydore STP did not meet all of the requirements in the EPA (NSW) guidelines (1997) [8] for use as a Grade A soil conditioner. Levels of both viruses and helminths were reduced through the solar drying treatment to acceptable levels. With regard to bacterial indicators, the results of this study indicate that *Salmonella* sp. and *E. coli* counts were reduced to acceptable levels for Grade A compliance. However, the results for the bacterial pathogens, particularly fecal coliforms, were inconclusive, primarily due to the chromogenic medium based rapid screening technique being unsuitable for monitoring environmental biosolids samples. The high diversity of microorganisms found in biosolids tended to confound the results although there was an overall reduction in potentially pathogenic microbes following the solar dryer process, but further testing would be required to quantify this reduction.

Organic chemical contaminants were found to be low but the high levels of several heavy metals was the main reason for the biosolids failing to meet the EPA (NSW) Guidelines (1997) [8] for use as a soil conditioner. Dilution of the biosolids, through the addition of low metal content, organic waste would be a relatively simple method to help solve this problem whereas extraction of the metals from the sludge would be an alternative but costly operation.

A rapid screening method for the detection of pathogens would be advantageous, but may not be available for some time given the complex nature of the sludge matrix. Intensive sampling using a rigorous experimental design combined with comprehensive and accurate methodologies for pathogen characterization would lead to a better understanding of the scientific processes underpinning the die-off kinetics of pathogens throughout the treatment process. Such a detailed, intensive procedure would not be feasible for compliance monitoring in the longer term. However, it could lead to the identification of critical control points during the solar drying treatment process (steps at which control can be applied to reduce specified hazards to acceptable levels). If critical limits for control parameters could then be defined which separate acceptability from unacceptability, the need for continuous characterization of pathogen levels would be diminished. An additional composting step at the end of the solar drying beds would constitute an example of such a critical control point, where sufficiently high temperatures could be achieved and maintained for the length of time required for pathogen die-off. This type of strategy for managing risks would complement ongoing efforts in developing reliable and feasible indicators for pathogens and other contaminants within biosolids and would guide management options.

Based on the present study, a number of recommendations have been made including dilution of biosolids with organic waste, addition of composting step, that could improve the operation of the solar dryer and lead to the improved likelihood of the treated biosolids meeting health-based criteria, possibly to the point where they can be classified for unrestricted use as a soil conditioner.

## Figures and Tables

**Table 1.** Classification of biosolids products based on contaminant and stabilization grades*.

Classification of Biosolids Products			
Biosolids Classification	Allowable Land Application Use	Minimum Quality Grades	
		Contaminant Grade	Stabilization Grade
Unrestricted Use	Home lawns and gardens Public contact sites Urban landscaping Agriculture Forestry Soil and site rehabilitation Landfill disposal Surface land disposal^2^	A	A
Restricted Use 1	Public contact sites Urban landscaping Agriculture Forestry Soil and site rehabilitation Landfill disposal Surface land disposal^2^	B	A
Restricted Use 2	Agriculture Forestry Soil and site rehabilitation Landfill disposal Surface land disposal^2^	C	B
Restricted Use 3	Forestry Soil and site rehabilitation Landfill disposal Surface land disposal^2^	D	B
Not suitable for use	Landfill disposal Surface land disposal^2^	E^1^	C^1^

1 Biosolids products which are not contaminant or stabilization graded are automatically classified as “Not Suitable for Use”.

2 To be applied within the boundaries of sewage treatment plant site.

*: EPA (NSW) “Environmental Guidelines: Use and Disposal of Biosolids Products” (1997) [8].

**Table 2.** Main processes conducted at the Maroochydore STP as part of the sludge treatment process stream.

Sludge Treatment Process Stream
Fermentation of primary sludge Thickening of waste fermented sludge using rotary screen thickeners Thickening of waste activated sludge (WAS) using dissolved air flotation (DAF) Anaerobic digestion of the thickened primary and secondary sludge Dewatering of the digested sludge using a centrifuge Solar drying of the dewatered sludge cake

**Table 3.** Biological standards for initial process verification and stabilization for Grade A biosolids*.

Initial Process Verification Standards	

Parameter	Standard
Enteric viruses	<1 PFU^a^ per 4 grams total dry solids
Helminth ova (*Ascaris* sp. and *Taenia* sp.)	<1 ovum per 4 grams total dry solids

Stabilization Grade A Microbiological Standards	

*Escherichia coli*	<100 MPN^b^ per gram (dry weight)
Fecal coliforms	<1 000 MPN per gram (dry weight)
*Salmonella* sp.	Not detected/50 grams of final product (dry weight)

a PFU: plaque forming unit

b MPN: most probable number

*: EPA (NSW) “Environmental Guidelines: Use and Disposal of Biosolids Products” (1997) [8].

**Table 4.** Contaminant acceptance concentration threshold levels for biosolid grades* (EPA (NSW) 1997).

Contaminant Acceptance Concentration Thresholds				
Contaminant	Grade A (mg/kg)	Grade B (mg/kg)	Grade C (mg/kg)	Grade D (mg/kg)
Arsenic	20	20	20	30
Cadmium	3	5	20	32
Chromium (total)	100	250	500	600
Copper	100	375	2,000	2,000
Lead	150	150	420	500
Mercury	1	4	15	19
Nickel	60	125	270	300
Selenium	5	8	50	90
Zinc	200	700	2,500	3,500
DDT/DDD/DDE	0.5	0.5	1.00	1.00
Aldrin	0.02	0.2	0.5	1.00
Dieldrin	0.02	0.2	0.5	1.00
Chlordane	0.02	0.2	0.5	1.00
Heptachlor	0.02	0.2	0.5	1.00
HCB	0.02	0.2	0.5	1.00
Lindane	0.02	0.2	0.5	1.00
BHC	0.02	0.2	0.5	1.00
PCBs	0.3	0.3	1.00	1.00

*: Grade A, unrestricted use; Grade B, restricted use 1 (public contact sites); Grade C, restricted use 2 (agriculture); Grade D, restricted use 3 (forestry).

**Table 5.** Indicator analyses for the biosolid samples collected from the *Maroochydore Sewage Treatment Plant.*

Initial Process verification Standard				

Parameter		Maroochy STP biosolids		

		Sample Run I Day 12	Sample Run II Day 1	Sample Run II Day 18

	Standard		Pre-treatment	Post-treatment
Viruses	<1 pfu/4 g total dry solids			
Enteric virus				<1 pfu/10 g
Adenovirus			95 pfu /10 g	<1 pfu/10 g
Enterovirus			32 pfu /10 g	<1 pfu/10 g
Reovirus			<1 pfu/10 g	<1 pfu/4 g

Bacteriophage		<1 pfu/4 g	>1 pfu/4 g	<1 pfu/4 g
Helminth ova	<1 ovum/4g total dry solids			
*Acaris* sp.		<1 ovum/10 g	-	<1 ovum/10 g
*Taenia* sp.		<1 ovum/10 g	-	<1 ovum/10 g

-: not detected.

**Table 6.** Presence/absence of microbial indicators in biosolids samples collected from the *Maroochydore Sewage Treatment Plant.*

Stabilization Grade A Microbiological Standards				

Parameter	Maroochydore STP Biosolids			

	Sample Run I Day 12 cfu/g dry weight of biosolids		Sample Run I Day 18 cfu/g dry weight of biosolids	

	MacConkey agar	*Salmonella/Shigella* agar	MacConkey	*Salmonella/Shigella* agar
*Escherichia coli*	-	-	-	-
Fecal coliforms	1.7 × 10^7^	2.2 × 10^7^	6.8 × 10^8^	6.8 × 10^8^
*Salmonella* sp.	-	-	-	-

- : not detected.

**Table 7.** Chromogenic identification of isolates on UriSelect®4 medium from final biosolid product.

Parameter	Maroochydore STP biosolids	

	Sample Run I: Day 12 cfu/g dry biosolids	Sample Run II: Day 18 cfu/g dry biosolids
*Candida albicans*	1.1 × 10^4^	2.9 × 10^6^
*Enterobacter cloacae*	9.6 × 10^5^	-
*Enterococcus fecalis*	5.8 × 10^5^	-
*Klebsiella pneumoniae*	-	-
*Proteus mirabilis*	9.6 × 10^4^	-
*Pseudomonas aeruginosa*	-	4.8 × 10^5^
*Staphylococcus aureus*	1.1 × 10^6^	-
*Staphylococcus saprophyticus*	-	-
*Streptococcus agalactiae*	2.2 × 10^6^	-

-: not detected.

**Table 8.** Percentage similarity of bacterial species identified using biochemical characterization of the isolates obtained from Uriselect®4 medium.

Strain code	Species isolated on UriSelect®4	Biochemically identified species	% similarity
U211–217	*Candida albicans*	*Cedecea davisae*	28.6
		*Hafnia alvei*	28.6
		*Klebsiella terrigena*	14.3
		*Rahnella aquatilis*	14.3
		*Serratia odorifera*	14.3
U221–227	*Enterobacter cloacae*	*Enterobacter pyrinus*	14.3
		*Hafnia alvei*	28.6
		*Klebsiella terrigena*	42.9
		*K. pneumoniae/S. sonnei^a^*	14.3
U231–237	*Enterococcus fecalis*	*Hafnia alvei*	28.6
		*Klebsiella terrigena*	71.4
U241–247	*Escherichia coli*	*Cedecea davisae*	28.6
		*Klebsiella pneumoniae^b^*	14.3
		*Klebsiella terrigena*	28.6
		*Morganella morganii^c^*	14.3
		*Providencia alcalifaciens*	14.3
U251–257	*Klebsiella pneumoniae*	*Cedecea davisae*	42.9
		*Klebsiella oxytoca*	14.3
		*Pantoea dispersa*	14.3
		*Providencia alcalifaciens*	14.3
		*O. proteus/T. guamensis^d^*	42.9
U261–267	*Proteus mirabilis*	*Cedecea davisae*	71.4
		*O. proteus/T. guamensis^d^*	28.6
U271–277	*Pseudomonas aeruginosa*	*Providencia alcalifaciens*	85.7
		*Yersinia enterocolitica*	14.3
U281–287	*Staphylococcus aureus*	*Cedecea davisae*	14.3
		*Klebsiella pneumoniae^b^*	28.6
		*O. proteus/T. guamensis^d^*	28.6
		*Providencia rustigianii*	14.3
		*Xenorhabdus nematophilus*	14.3
U291–297	*Streptococcus agalactiae*	*Cedecea davisae*	42.9
		*Enterobacter cancerogenus^e^*	14.3
		*O. proteus/T. guamensis^d^*	28.6
		*Xenorhabdus nematophilus*	14.3

a *Klebsiella pneumoniae* (subsp. *rhinoscleromatis*); *Shigella sonnei*,

b *Klebsiella pneumoniae* (subsp. *pneumoniae*),

c *Morganella morganii* (subsp. *sibonii*),

d *Obesumbacterium proteus* biogroup 2; *Tatumella ptyseos*.

e Refer to *Enterobacter aerogenes* for 2^nd^-most-likely possibility (Cappuccino & Sherman, 2008) [27].

**Table 9.** Sensitivity of biosolids-derived isolates to various antibiotics.

Isolate code	*Cedecea davisae U243*	*Enterobacter cancerogenus S201*	*Hafnia alvei M203*	*Klebsiella oxytoca U257*	*Klebsiella terrigena U235*	*Pantoea dispersa U256*	*Providencia alcalifaciens U245*	*Serratia odorifera M104*
Antibiotics								
Ampicillin 10 μg	–	–	–	–	+	–	+	–
Cephalothin 30 μg	–	+	+	–	+	–	+	+
Chloramphenicol 30 μg	+	+	+	±	+	±	+	+
Ciprofloxacin 5 μg	+	+	+	±	+	+	+	+
Clindamycin 2 μg	–	–	–	–	+	–	–	–
Doxycycline 30 μg	±	+	+	–	+	+	+	+
Erythromycin 15 μg	+	+	–	–	–	–	±	±
Gentamicin 10 μg	–	–	±	–	+	–	+	±
Kanamycin 30 μg	–	+	+	–	–	+	+	+
Methicillin 5 μg	–	–	–	–	+	–	+	–
Nalidixic acid 30 μg	±	+	+	–	–	–	+	+
Nitrofurantoin 300 μg	–	+	+	–	+	–	+	+
Penicillin G 10 μg	–	–	–	–	+	–	+	–
Rifampicin 5 μg	–	–	+	–	+	–	+	–
Streptomycin 10 μg	±	–	+	–	+	–	+	+
Sulphafurazole 300 μg	±	–	–	–	–	–	±	+
Tetracycline 30 μg	–	±	±	–	+	+	+	+
Trimethoprim 5 μg	–	–	+	±	–	–	+	–
Trimethoprim 1.25 μg/Sulfamethoxazole 25.75 μg	+	+	+	–	–	–	+	–

+: sensitive

–: resistant

±: Intermediate

**Table 10.** Metal/metalloid concentrations in final biosolids product together with threshold concentrations defined in the guidelines*.

Metal/Metalloid Concentrations		

Contaminant	Maroochydore STP Biosolids (mg/kg)	

	Sample I: Day 12	Sample II: Day 18
Arsenic	8 (A)	9 (A)
Cadmium	2 (A)	2 (A)
Chromium (total)	27 (A)	29 (A)
Copper	402 (C)	406 (C)
Lead	19 (A)	18 (A)
Mercury	0.8 (A)	1.1 (B)
Nickel	22 (A)	23 (A)
Selenium	<5 (A)	<5 (A)
Zinc	620 (B)	649 (B)

Grade A, unrestricted use; Grade B, restricted use 1 (public contact sites); Grade C, restricted use 2 (agriculture); Grade D, restricted use 3 (forestry) (refer to Table 10 for Contaminant Acceptance Concentration Thresholds).

*: EPA (NSW) “Environmental Guidelines: Use and Disposal of Biosolids Products” (1997) [8].
